# Management of a Pneumothorax Found Intraoperatively During Emergent Surgery

**DOI:** 10.7759/cureus.88460

**Published:** 2025-07-21

**Authors:** Logan A Goddard, Mitchell Hueniken, Ivana Ezeude, Gisele J Wakim

**Affiliations:** 1 Anesthesiology, University of Miami Miller School of Medicine, Miami, USA; 2 Anesthesiology, Jackson Memorial Hospital, Miami, USA

**Keywords:** anesthesia, chest tube, emergent surgery, intraoperative pneumothorax, tension pneumothorax

## Abstract

Pneumothorax is a potentially life-threatening condition that occurs when air enters the pleural cavity. There are numerous causes of pneumothorax, and healthcare providers must identify and treat the condition quickly to avoid elevated morbidity and mortality.

This report describes the case of an 87-year-old man with several comorbidities and a chronic tracheostomy on a ventilator who presented to the emergency department (ED) with bleeding at the tracheostomy site. Initial workup by otolaryngology (ENT) revealed hemostasis at the tracheostomy site and a retrocardiac opacity suspicious for pneumonia, so he was admitted to the intensive care unit (ICU). The patient was later noted to have oozing at the tracheostomy site and difficult ventilation, and a bronchoscopy revealed clots in the trachea. Ventilation became increasingly difficult, and the patient went into cardiopulmonary arrest, requiring chest compressions and defibrillation. After clot removal and spontaneous circulation was achieved, the patient continued to have persistent desaturations and was taken to the operating room (OR) by ENT for surgical exploration of the upper airway. During the surgery, two intraoperative chest X-rays (CXRs) taken 1.5 hours apart revealed a right pneumothorax. The surgeon opted to postpone chest tube insertion until after the procedure, and the pneumothorax was successfully decompressed in the medical intensive care unit (MICU) after the completion of the surgery. The patient returned to baseline health.

Our report demonstrates an uncommon case of pneumothorax discovered intraoperatively during emergent surgery. Pneumothoraces can cause rapid deterioration of hemodynamic stability and need to be identified quickly by providers. In the context of risk factors such as recent chest compressions, physicians should maintain high clinical suspicion for pneumothorax.

## Introduction

A pneumothorax occurs when air enters the pleural space, an area between the lungs and the chest wall. This accumulation of air between the visceral and parietal pleura creates pressure on the lung, causing it to collapse partially or completely. This can lead to symptoms such as sudden chest pain, dyspnea, and tachycardia. 

There are three major causes of a pneumothorax: primary spontaneous, secondary spontaneous, and traumatic. A primary spontaneous pneumothorax occurs in the absence of known lung disease; although the direct cause of these is unknown, risk factors such as smoking, male sex, and family history are associated with its development [1]. A secondary spontaneous pneumothorax occurs due to underlying lung diseases, with approximately 70% of these cases occurring in patients with chronic obstructive pulmonary disease (COPD) [1]. Lastly, a traumatic pneumothorax occurs when the chest wall is pierced, usually by stab wounds, gunshot wounds, or rib fractures. Traumatic pneumothoraces develop about 10% of the time as a complication of chest compressions during cardiopulmonary resuscitation (CPR), with about half of these cases progressing to a tension pneumothorax, especially if positive pressure ventilation is applied [2,3]. 

Regardless of the etiology of a pneumothorax, the condition can progress and increase in size to the point where hemodynamic stability is compromised. A tension pneumothorax occurs when the collapse of one lung is so severe that it results in a mediastinal shift that compresses the contralateral lung, thus leading to decreased cardiac output, increased pulmonary vascular resistance, and even circulatory collapse and cardiac arrest [3].

## Case presentation

An 87-year-old male patient with a past medical history significant for complete atrioventricular (AV) block with permanent pacemaker (on apixaban), obstructive sleep apnea, obesity, hypoventilation on CPAP at night, hypertension, and prior deep vein thromboses (DVT) living in a long-term facility on a ventilator with chronic tracheostomy presented to the emergency department (ED) for bleeding at the tracheostomy site. Otolaryngology (ENT) was consulted and found no further bleeding at the tracheostomy site. An initial chest X-ray (CXR) in the ED showed a retrocardiac opacity suspicious for pneumonia. Apixaban was stopped, and the patient was admitted to the intensive care unit (ICU) for management of his pneumonia.

A CXR in the ICU three days later showed emphysematous changes, mild pulmonary edema, no pneumothorax, and an improved retrocardiac opacity likely representing atelectasis. A CXR was repeated the following day with no significant changes and no pneumothorax present. Three days later, the patient was noted to have large clots and active oozing around the tracheostomy site, with poor ventilation, low volume, and high peak airway pressures. The patient was manually ventilated and underwent bronchoscopy by the ENT and ICU attending at the bedside in the ICU. Large clots were found almost completely occluding the trachea and proved difficult to remove. It became increasingly challenging to ventilate, leading to a cardiopulmonary arrest with an initial rhythm of pulseless electrical activity, followed by ventricular tachycardia and ventricular fibrillation. The patient underwent chest compressions and was defibrillated three times. Eventually, a large clot was removed from the airway, and return of spontaneous circulation (ROSC) was achieved while still in the ICU. The total time between cardiac arrest and ROSC was 18 minutes. Norepinephrine (10.5 mcg/kg/hour) and epinephrine drips (1.05 mcg/kg/hour) were utilized to maintain a mean arterial pressure (MAP) >65 mmHg in accordance with current guidelines [4]. Over the course of these events, an endotracheal tube (ETT) was placed at the tracheostomy site, and the patient was taken to the operating room (OR) for emergent surgical upper airway exploration and tracheostomy tube placement with ENT.

Upon arrival in the OR, standard monitors and an arterial line were placed on the left dorsalis pedis artery. Initial ventilator settings were volume-control/assist-control with a respiratory rate (RR) of 15, positive end-expiratory pressure (PEEP) of 5 cm H_2_O, tidal volume (TV) of 475 mL (5.6 mL/kg), and 40% fraction of inspired oxygen (FiO_2_). Ventilator settings during and immediately after the case were in volume-control ventilation mode with an RR of 18, PEEP of 5 cm H_2_O, TV of 460 mL (5.4 mL/kg), and 100% FiO_2_. The initial arterial blood gas was significant for a respiratory acidosis and hypoxemia (Table 1). Other signs of inadequate ventilation at this time were tachycardia, increased peak airway pressures, and peripheral oxygen saturation (SpO_2_) ranging from 77% to 90%. Given these signs, in addition to auscultation of decreased bilateral breath sounds and a recent history of chest compressions, there was a high clinical suspicion for a pneumothorax, and an intraoperative CXR was ordered.

**Table 1:** ABG data throughout surgery ABG: Arterial blood gas; tHb: Total hemoglobin; HCO_3_^-^: Bicarbonate; Na^+^: Sodium; K^+^: Potassium; Cl^-^: Chloride; Ca^++^: Calcium; HCT: Hematocrit; Glu: Glucose; SO_2_: Oxygen saturation; PO_2_: Partial pressure of oxygen; PCO_2_: Partial pressure of carbon dioxide

Parameter	Initial ABG	20 min later	20 min later	Final ABG	Reference range	Unit
pH	7.23	7.37	7.38	7.4	7.35-7.45	-
PCO_2_, arterial	88.7	44	46	43	35-45	mmHg
HCO_3_^-^, arterial	27.6	25.4	27.2	26.6	22-26	mmHg
PO_2_	-	55	59	63	80-100	mmHg
SO_2_	88.7	88.2	92.4	-	95-100	%
Na^+^	148	159	147	148	135-145	mEq/L
K^+^	4.2	4.5	4.2	4.1	3.5-5.0	mEq/L
Cl^-^	112	111	112	111	95-105	mEq/L
Ca^++^	1.82	1.64	1.52	1.51	8.4-10.2	mg/dL
HCT	27	26	24	23	36-44	%
Glu	188	181	184	183	<140	mmol/L
Lactate	38	29	21	18	4.5-19.8	mg/dL
tHb	8.5	8.3	8.1	7.9	13-18	g/dL

**Figure 1:**
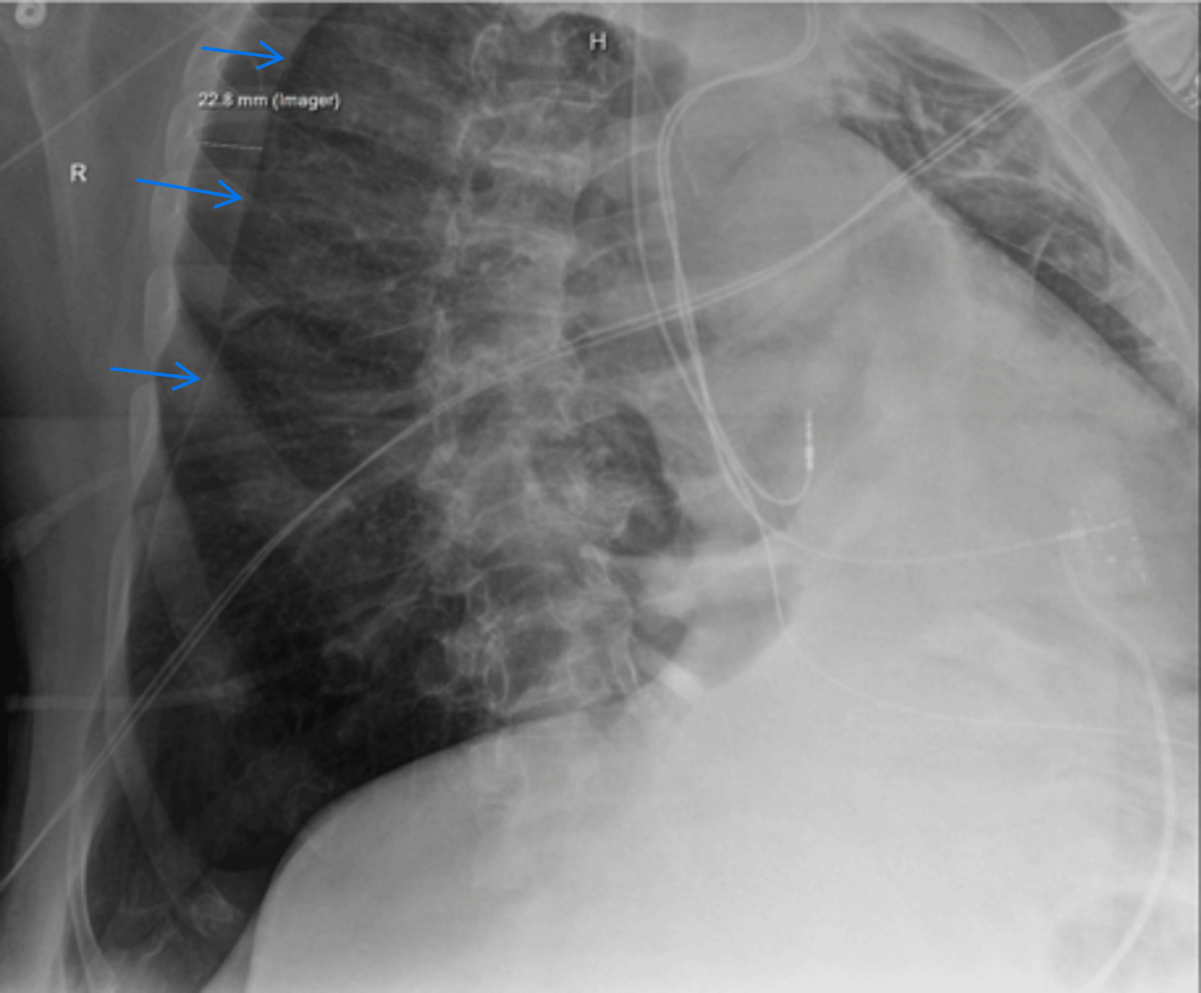
Intraoperative chest X-ray #1 Arrows show right-sided pneumothorax.

**Figure 2:**
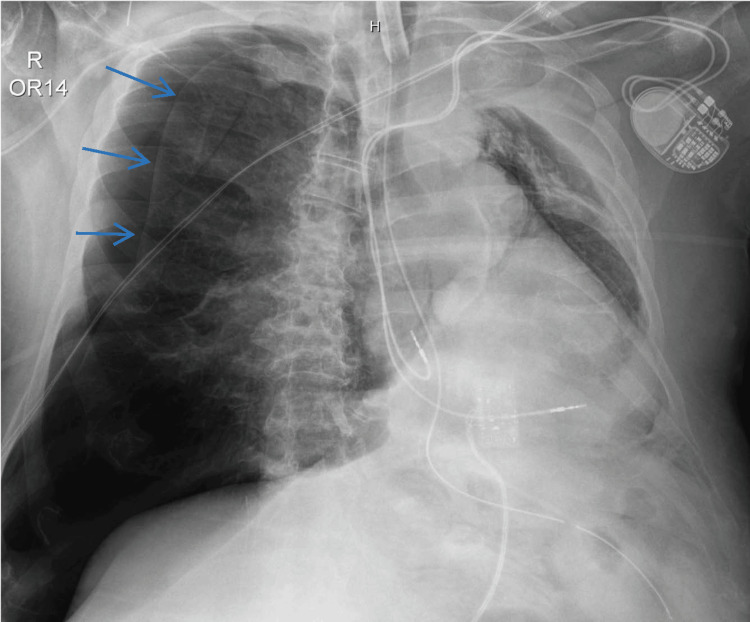
Intraoperative chest X-ray #2 Arrows show right-sided tension pneumothorax, initially read as stable and then subsequently updated to expanding.

The CXR was read as showing a moderate right-sided pneumothorax (with an intrapleural distance laterally of 2.3 cm) and a large left pleural effusion (Figure 1). Mediastinal shift could not be ruled out due to patient positioning. Given these findings, cardiothoracic surgery was consulted for chest tube placement; however, the surgeon declined, opting rather to place the chest tube after the surgery. Although the patient’s blood gases improved throughout the case (Table 1), the patient remained difficult to ventilate. One hour into the case, another intraoperative CXR was taken and again read by radiology as a moderately sized but stable and unchanged pneumothorax (Figure 2). The surgeons managed to exchange the ETT for a tracheostomy tube, remove blood clots, and perform a bronchoscopy to confirm the airway was clear with the tracheostomy tube in the appropriate position.

**Figure 3:**
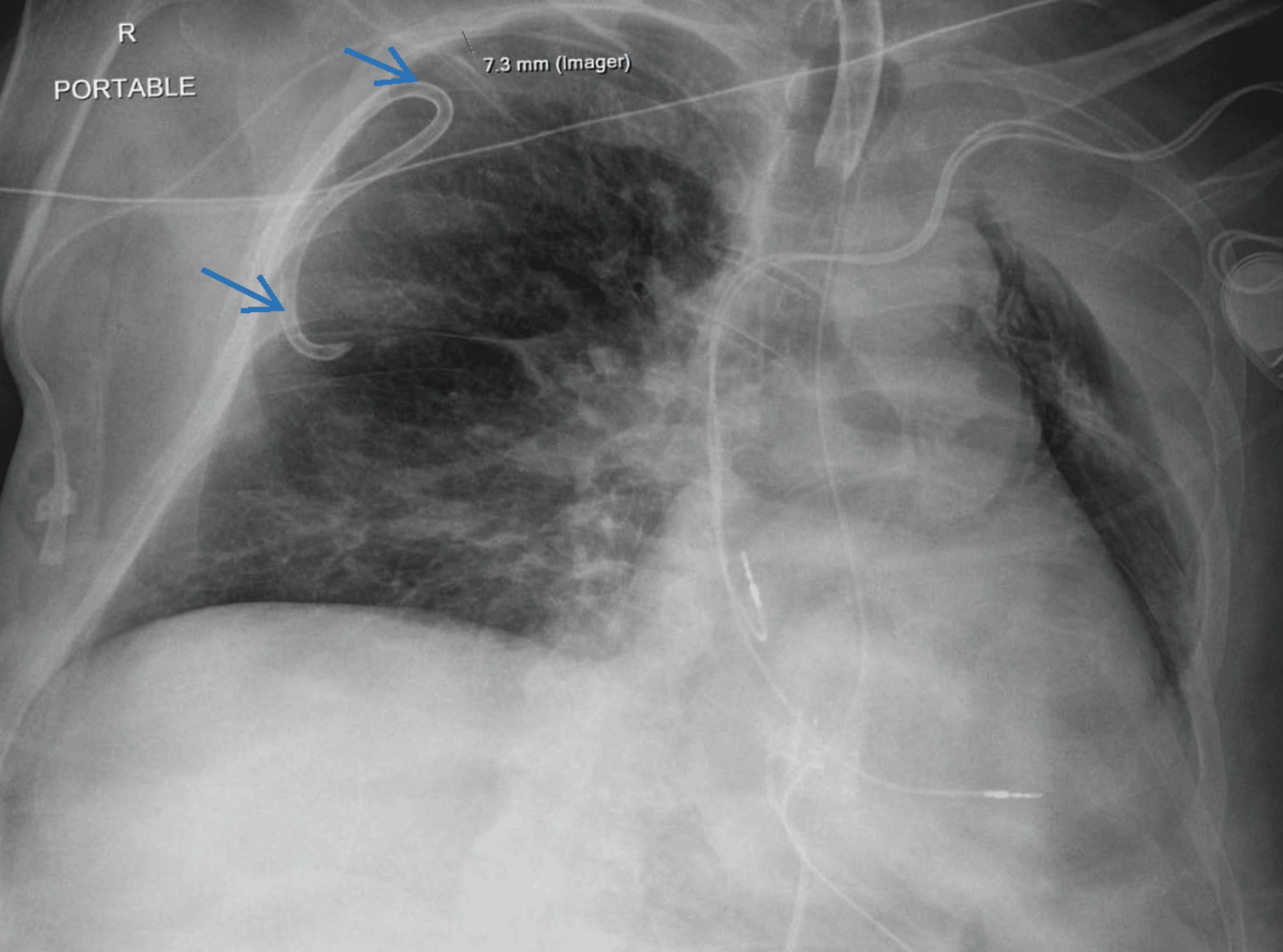
Postoperative chest X-ray Arrows show appropriate chest tube positioning and resolution of pneumothorax.

After the completion of the case, the patient was transferred to the medical intensive care unit (MICU) with the tracheostomy tube in place. Upon arrival at the MICU, the radiology attending notified the MICU of an updated reading on the second intraoperative CXR (Figure 2); an expanding right tension pneumothorax was visualized, and immediate needle decompression was recommended. The MICU attending promptly placed a chest tube and confirmed proper placement and resolution of the tension pneumothorax with a third CXR, taken 4.5 hours after the second intraoperative CXR (Figure 3). Subsequent computed tomography (CT) brain imaging revealed no evidence of ischemic injury, infarctions, or intracranial hemorrhages. The patient had an uneventful remainder of his inpatient course and fully returned to baseline.

## Discussion

CPR is associated with numerous chest injuries, including lung parenchymal injury, skeletal fractures, and pneumothorax [5]. Several studies estimate that in patients receiving chest compressions, the incidence of sternal fractures is 72%, while pneumothorax occurs 9-11% of the time [2,5]. Due to the high rates of complications, most hospitals require chest imaging after successful ROSC to rule out iatrogenic trauma. In this case, the patient was brought emergently to the OR after ROSC, with no time for radiologic assessment. The lack of chest imaging prior to anesthesia presented a high risk, given the known complications of anesthesia and positive pressure ventilation in patients with even a small preexisting pneumothorax [6-8]. 

Once in the OR, an intraoperative CXR was expediently ordered and showed a right-sided pneumothorax. At this point, several mistakes were made. Primarily, the pneumothorax should have been immediately decompressed, despite the surgeon’s preference to wait until after the completion of surgery. Continued positive pressure ventilation was extremely risky, given the presence of moderate pneumothorax, and likely contributed to the development of a tension pneumothorax [8]. Furthermore, the second intraoperative CXR that was ordered to monitor the pneumothorax was misread by radiology. It was initially read as stable, but an updated read postoperatively identified an expanding tension pneumothorax. If the tension pneumothorax had been correctly identified during the first read, the surgeon would likely have agreed to immediate decompression rather than continuing to delay intervention.

However, CXR frequently fails to identify tension physiology, particularly in supine positioning. According to the Society for Critical Care Medicine, the sensitivity of CXR for detecting any pneumothorax is 30-75%, with sensitivity for tension pneumothorax even lower [9]. While the precise sensitivity for tension pneumothorax is not well documented, there is consensus that it is below 50% [10-12]. Ultrasound could have been performed, as it has higher sensitivity for detecting pneumothorax; however, its sensitivity for detecting tension physiology is highly variable [12]. Given that the pneumothorax was already recognized on CXR, the utility of ultrasound for further characterization was quite limited. Ultimately, CT is the gold standard for diagnosing both pneumothorax and tension pneumothorax. CT improves sensitivity to nearly 100%, but is logistically impossible to perform intraoperatively and thus would not have proved useful in this case [9].

## Conclusions

Early diagnosis and management of a suspected intraoperative pneumothorax is imperative, as patients can quickly decompensate and develop a tension pneumothorax due to the application of positive pressure ventilation during surgery. Even when there is evidence that the pneumothorax does not display tension physiology, decompression should still be performed immediately. In this case, delays in decompression due to surgeon preference and radiology misreads led to a tension pneumothorax. Fortunately, the patient did not deteriorate and remained hemodynamically stable postoperatively, making a full return to his baseline in the days that followed.
